# Closing the critical care knowledge gap: the importance of publications from low-income and middle-income countries

**DOI:** 10.62675/2965-2774.20240251ed-en

**Published:** 2024-11-14

**Authors:** Jorge Ibrain Figueira Salluh, Bruno Adler Maccagnan Pinheiro Besen, Sebastián González-Dambrauskas, Suchitra Ranjit, Daniela Carla Souza, Viviane Cordeiro Veiga, Mervyn Mer, Alejandro Bruhn, Otavio T. Ranzani, Luigi Pisani, Diptesh Aryal, Madiha Hashmi, Sheila Nainan Myatra, Juliana Carvalho Ferreira, Antonio Paulo Nassar

**Affiliations:** 1 Instituto D’Or de Pesquisa e Ensino Rio de Janeiro RJ Brazil Instituto D’Or de Pesquisa e Ensino - Rio de Janeiro (RJ), Brazil.; 2 Universidade de São Paulo Hospital das Clínicas Faculdade de Medicina São Paulo SP Brazil Hospital das Clínicas, Faculdade de Medicina, Universidade de São Paulo - São Paulo (SP), Brazil.; 3 Universidad de la República Facultad de Medicina Centro Hospitalario Pereira Rossell Montevideo Uruguay Departamento de Pediatría y Unidad de Cuidados Intensivos de Niños, Centro Hospitalario Pereira Rossell, Facultad de Medicina, Universidad de la República - Montevideo, Uruguay.; 4 Apollo Children's Hospital Pediatric Intensive Care and Emergency Services Chennai India Pediatric Intensive Care and Emergency Services, Apollo Children's Hospital - Chennai, India.; 5 Universidade de São Paulo Hospital Universitário Department of Pediatrics São Paulo SP Brazil Department of Pediatrics, Hospital Universitário, Universidade de São Paulo - São Paulo (SP), Brazil.; 6 BP - A Beneficência Portuguesa de São Paulo São Paulo SP Brazil BP - A Beneficência Portuguesa de São Paulo - São Paulo (SP), Brazil.; 7 Charlotte Maxeke Johannesburg Academic Hospital Divisions of Critical Care and Pulmonology Department of Medicine Johannesburg South Africa Department of Medicine, Divisions of Critical Care and Pulmonology, Charlotte Maxeke Johannesburg Academic Hospital - Johannesburg, South Africa.; 8 Pontificia Universidad Católica de Chile Facultad de Medicina Departamento de Medicina Intensiva Santiago Chile Departamento de Medicina Intensiva, Facultad de Medicina, Pontificia Universidad Católica de Chile - Santiago, Chile.; 9 Universidade de São Paulo Faculdade de Medicina Hospital das Clínicas São Paulo SP Brazil Instituto do Coração, Hospital das Clínicas, Faculdade de Medicina, Universidade de São Paulo - São Paulo (SP), Brazil.; 10 University of Bari "Aldo Moro" Section of Anesthesiology and Intensive Care Medicine Department of Precision-Regenerative Medicine and Jonic Area Bari Italy Department of Precision-Regenerative Medicine and Jonic Area, Section of Anesthesiology and Intensive Care Medicine, University of Bari "Aldo Moro" - Bari, Italy.; 11 Nepal Intensive Care Research Foundation Kathmandu Nepal Nepal Intensive Care Research Foundation - Kathmandu, Nepal.; 12 Ziauddin University Department of Critical Care Medicine Karachi Pakistan Department of Critical Care Medicine, Ziauddin University - Karachi, Pakistan.; 13 Homi Bhabha National Institute Tata Memorial Hospital Department of Anesthesiology, Critical Care and Pain Mumbai India Department of Anesthesiology, Critical Care and Pain, Tata Memorial Hospital, Homi Bhabha National Institute - Mumbai, India.; 14 A. C. Camargo Cancer Center Intensive Care Unit São Paulo SP Brazil Intensive Care Unit, A. C. Camargo Cancer Center - São Paulo (SP), Brazil.

The burdens of critical illness and adequate access to care remain disproportionately high in low- and middle-income countries (LMICs). The burden and outcomes mirror socioeconomic factors such as Gross Domestic Product (GDP) and health care spending, extending to critical care research and publications.^(1)^ High-income countries (HICs) dominate the research landscape, overlooking the unique challenges faced by LMICs. This imbalance is a matter of inequality that imposes a barrier to improving health outcomes. Clinical trial activity, both researcher- and sponsored-initiated, is often not aligned with this regional disease burden, resulting in a mismatch between disease frequency and the target population of clinical trials.^(2)^

The global burden of critical illness and its consequences are strikingly unequal, reflecting disparities in disease burden and human and technological resources. This includes intensive care unit (ICU) beds and well-trained providers. While HICs have an average of 34.8 ICU beds per 100,000 people, middle-income countries (MICs) have an average of 13.3 ICU beds per 100,000 inhabitants, and low-income countries (LICs) only have an average of 3.1 ICU beds per 100,000 inhabitants according to the Organization for Economic Cooperation and Development (OECD; www.oecd.org). Low- and middle-income countries also have a significant shortage of ICU nurses, with data indicating that the nurse-to-patient ratio (a well-recognized element of high-quality ICU care) is very distant from that of their HIC counterparts.^(3)^ The coronavirus disease 2019 (COVID-19) pandemic distinctly illustrates these disparities, with LMICs facing limited access to critical care alongside a shortage of well-trained staff. While nurse shortages have been a longstanding reality in LMICs, they have also recently affected HICs due to the pandemic. Therefore, studying solutions for this issue in LMICs may soon benefit HICs as well.^(4,5)^

The need for LMIC-based research is paramount and driven by differences in epidemiology, health systems, outcomes, practices, and infrastructure. The epidemiology and outcomes of critical illnesses in LMICs can differ vastly from those in HICs. Practices and structures within ICUs also vary significantly. The quantity and degree of specialization of health care personnel in LMICs remain suboptimal. This disparity may affect the applicability of research findings for the management of critical illness-related conditions. The process of translating knowledge from HICs to LMICs is fraught with challenges. While some conclusions may be generalizable, many are not, in part owing to differences in ICU structures, case mixes, and health system characteristics. The effectiveness of an intervention in a well-staffed ICU in an HIC may not translate to a setting where health care workers are overburdened and resources are scarce.

Funding for research is a primary obstacle, as financial resources are often allocated to more immediate health care needs rather than research. The total health expenditure per capita in LMICs is often less than 1,000 US dollars (USD) in MICs and 100 USD in LICs, whereas it is more than 4,000 USD in HICs. Additionally, the shortage of trained personnel to design, conduct, and analyze research studies hinders the development of locally relevant research that can inform practice and policy.

Addressing these disparities requires a concerted effort to support LMIC-based research (Figure 1). Increasing funding, building local research capacity, and fostering international collaboration are essential steps. There are several successful examples from other areas of medicine (i.e., HIV, tuberculosis) in LMICs that have led to sustainable funding and capacity building. The Africa Health Research Institute (https://www.ahri.org/) and the INDEPTH Network (https://www.indepth-network.org/) are examples of initiatives committed to decolonizing health research. Intensive care research networks are transformative and thriving movements where researchers from both HICs and LMICs can collaborate globally and mutually benefit, ultimately demonstrating that collective efforts surpass individual contributions. By amplifying the voices and expertise of researchers in LMICs, we can ensure that the global critical care community benefits from diverse insights and solutions tailored to different contexts. A key point for success is to adopt principles raised by the #DecolonizeScience movement, guaranteeing that Global South researchers and partners have a real seat at the table, from the design and prioritization of the research question to authorship. The amount of funding allocated to LMIC institutions is crucial in this process, covering not only the study but also the training of local staff and aiming to leave in that country improved understanding and skills in terms of leadership for research.

**Figure 1:**
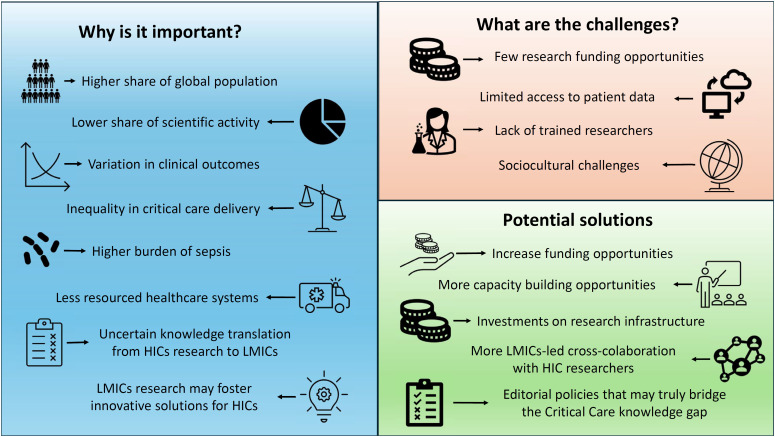
Importance, challenges and potential solutions for overcoming the critical care knowledge gap in low-income and middle-income countries.

LMICs - low- and middle-income countries; HICs - high-income countries.

## PUBLICATION BARRIERS, BIASES AND "OPEN ACCESS": THE ROLE OF JOURNALS IN MITIGATING INEQUALITIES AND ENHANCING RESEARCH PUBLICATION OUTPUTS FROM LOW- AND MIDDLE-INCOME COUNTRIES

Despite housing 84% of the world's population, there is substantial underrepresentation of research from LMICs.^(6)^ This occurs through a low or negligible proportion of patients from LMICs in large international studies, a small number of studies conducted primarily in LMICs or by having exclusively LMIC studies where HIC-based researchers and institutions drive the research question and intellectual work. This creates a large knowledge gap that stems from several challenges. First, the dominance of English-language journals creates a barrier for nonnative speakers in HIC-based journals, in a world where a fraction of the population is native English speakers.^(7)^ Second, publication costs pose a significant barrier, as they are a major burden for researchers in LMICs with limited overall budgets and, with little or none available for publication fees.^(8)^ Although there are initiatives, such as waiving article processing charges for LICs, this excludes MICs. Inequality is evident in both the ability to fund research and to fund its publication in a scenario where "open access" is increasingly common as it is associated with high publication costs. Finally, evidence suggests a bias in the editorial and peer-review process,^(9)^ favoring studies from academic centers in HICs for publication in high-impact journals.^(10)^ These publication biases have further consequences. As artificial intelligence increasingly influences health care, its algorithms learn from existing data, potentially perpetuating these biases when applied to critical care in LMIC settings.

The full involvement of local researchers is fundamental and must be reflected in equal authorship positions. As researchers and medical journal editors, we play a vital role in mitigating publication inequities. Critical Care Science is committed to providing a platform for all countries, regardless of geography, language, or socioeconomic status. Our diverse editorial board and full open-access format (free for authors and readers) with the elimination of article processing charges contribute to this mission to create a safe haven for publications by researchers from LMICs while guaranteeing high-quality scientific standards. The traditional open-access model is intrinsically flawed. It may provide waivers for LICs and be open for readers worldwide but has high subscription costs for universities and academic centers in addition to the extremely high publication costs for MIC-based researchers.

Moving toward an unbiased publication system, with fair peer review and financially accessible scientific publications for all, should become a priority. Sliding scales or entirely free publications should be the goal. It requires a strong commitment from academia, medical societies, and funders. These are crucial steps toward closing the critical care knowledge gap and improving global health outcomes. This journal is taking a firm step in this direction. We encourage other journals to join Critical Care Science in this endeavor by adopting similar practices and actively promoting research from LMICs. By fostering a more equitable research landscape, we will ensure that the much-needed critical care advancements improve the quality of current evidence and accelerate its implementation internationally, ultimately improving care delivery and saving lives.
