# Supplementary material for: Closing the critical care knowledge gap: the importance of publications from low-income and middle-income countries
Source: Crit Care Sci. 2024 Nov 14;36:e20240251eden. doi: 10.62675/2965-2774.20240251ed-en (PMC11634232; doi:10.62675/2965-2774.20240251ed-en)
Supplement: Translation

# Translation: Preenchendo a lacuna de conhecimento em cuidados intensivos: a importância das publicações de países de baixa e média renda

**DOI:** 10.62675/2965-2774.20240251ed-pt

**Authors:** Jorge Ibrain Figueira Salluh, Bruno Adler Maccagnan Pinheiro Besen, Sebastián González-Dambrauskas, Suchitra Ranjit, Daniela Carla Souza, Viviane Cordeiro Veiga, Mervyn Mer, Alejandro Bruhn, Otavio T. Ranzani, Luigi Pisani, Diptesh Aryal, Madiha Hashmi, Sheila Nainan Myatra, Juliana Carvalho Ferreira, Antonio Paulo Nassar

**Affiliations:** 1 Rio de Janeiro RJ Brasil Instituto D’Or de Pesquisa e Ensino - Rio de Janeiro (RJ), Brasil.; 2 São Paulo SP Brasil Hospital das Clínicas, Faculdade de Medicina, Universidade de São Paulo - São Paulo (SP), Brasil.; 3 Montevideo Uruguai Departamento de Pediatría y Unidad de Cuidados Intensivos de Niños, Centro Hospitalario Pereira Rossell, Facultad de Medicina, Universidad de la República - Montevideo, Uruguai.; 4 Chennai India Pediatric Intensive Care and Emergency Services, Apollo Children's Hospital - Chennai, India.; 5 São Paulo SP Brasil Departamento de Pediatria, Hospital Universitário, Universidade de São Paulo - São Paulo (SP), Brasil.; 6 São Paulo SP Brasil BP - A Beneficência Portuguesa de São Paulo - São Paulo (SP), Brasil.; 7 Johannesburg South Africa Department of Medicine, Divisions of Critical Care and Pulmonology, Charlotte Maxeke Johannesburg Academic Hospital - Johannesburg, South Africa.; 8 Santiago Chile Departamento de Medicina Intensiva, Facultad de Medicina, Pontificia Universidad Católica de Chile - Santiago, Chile.; 9 São Paulo SP Brasil Instituto do Coração, Hospital das Clínicas, Faculdade de Medicina, Universidade de São Paulo - São Paulo (SP), Brasil.; 10 Bari Italia Department of Precision-Regenerative Medicine and Jonic Area, Section of Anesthesiology and Intensive Care Medicine, University of Bari "Aldo Moro" - Bari, Italia.; 11 Kathmandu Nepal Nepal Intensive Care Research Foundation - Kathmandu, Nepal.; 12 Karachi Pakistan Department of Critical Care Medicine, Ziauddin University - Karachi, Pakistan.; 13 Mumbai India Department of Anesthesiology, Critical Care and Pain, Tata Memorial Hospital, Homi Bhabha National Institute - Mumbai, India.; 14 São Paulo SP Brasil Unidade de Terapia Intensiva, A. C. Camargo Cancer Center - São Paulo (SP), Brasil.

O ônus das doenças graves e o acesso adequado aos cuidados permanecem desproporcionalmente altos nos países de baixa e média renda (PBMRs). O ônus e os desfechos refletem fatores socioeconômicos, como o Produto Interno Bruto (PIB) e os gastos com saúde, estendendo-se à pesquisa e às publicações sobre cuidados intensivos.^(1)^ Os países de alta renda (PARs) dominam o cenário da pesquisa, ignorando os desafios exclusivos enfrentados pelos PBMRs. Esse desequilíbrio é uma questão de desigualdade que impõe uma barreira à melhoria dos indicadores de saúde. A atividade de ensaios clínicos, tanto iniciada por pesquisadores quanto patrocinada, muitas vezes não está alinhada com essa problemática regional de doenças, resultando em uma incompatibilidade entre a frequência das doenças e a população-alvo dos ensaios clínicos.^(2)^

A incidência global de doenças graves e suas consequências são surpreendentemente desiguais, refletindo as disparidades entre a incidência de doenças e os recursos humanos e tecnológicos. Isso inclui leitos de unidade de terapia intensiva (UTI) e profissionais bem treinados. Enquanto os PARs têm média de 34,8 leitos de UTI por 100 mil habitantes, os países de renda média (PMRs) têm média de 13,3 leitos de UTI por 100 mil habitantes, e os países de baixa renda (PBRs) têm apenas média de 3,1 leitos de UTI por 100 mil habitantes, segundo a *Organization for Economic Cooperation and Development* (OCDE; www.oecd.org). Os PBMRs também têm uma escassez significativa de enfermeiros de UTI, com dados indicando que a proporção de enfermeiros por paciente (um elemento bem reconhecido dos cuidados de alta qualidade em UTIs) está muito distante da de seus colegas de PARs.^(3)^ A pandemia da doença pelo coronavírus 2019 (COVID-19) ilustra claramente essas disparidades, pois os PBMRs enfrentam acesso limitado aos cuidados críticos, além da escassez de pessoal bem treinado. Embora a escassez de enfermeiros seja uma realidade antiga nos PBMRs, ela também afetou recentemente os PARs devido à pandemia. Portanto, estudar soluções para esse problema em PBMRs pode, em breve, beneficiar também os PARs.^(4,5)^

A necessidade de pesquisas em PBMRs é primordial e impulsionada por diferenças na epidemiologia, nos sistemas de saúde, nos desfechos, nas práticas e na infraestrutura. A epidemiologia e os desfechos de doenças graves nos PBMRs podem ser muito diferentes daqueles dos PARs. As práticas e a estrutura das UTIs também variam significativamente. A quantidade e o grau de especialização da equipe de saúde nos PBMRs permanecem abaixo do ideal. Essa disparidade pode afetar a aplicabilidade dos achados de pesquisas para o manejo de condições relacionadas a doenças críticas. O processo de tradução do conhecimento dos PARs para os PBMRs é repleto de desafios. Embora algumas conclusões possam ser generalizáveis, muitas não o são, em parte devido às diferenças nas estruturas das UTIs, nas combinações de casos e nas características do sistema de saúde. A eficácia de uma intervenção em uma UTI com boa equipe em um PAR pode não se traduzir em um ambiente em que os profissionais de saúde estão sobrecarregados, e os recursos são escassos.

O financiamento da pesquisa é um grande obstáculo, já que os recursos financeiros costumam ser alocados em necessidades mais imediatas de cuidados com a saúde, em vez de serem destinados à pesquisa. O gasto total com saúde *per capita* nos PBMRs costuma ser inferior a US$ 1.000 nos PMRs e a US$ 100 nos PBRs, enquanto nos PARs é superior a US$ 4.000. Além disso, a falta de pessoal treinado para desenhar, conduzir e analisar estudos de pesquisa dificulta o desenvolvimento de pesquisas localmente relevantes que possam informar a prática e as diretrizes.

O enfrentamento dessas disparidades exige um esforço conjunto para apoiar a pesquisa em PBMRs (Figura 1). Aumentar o financiamento, desenvolver a capacidade de pesquisa local e promover a cooperação internacional são etapas essenciais. Há vários exemplos exitosos de outras áreas da medicina (a saber, HIV, tuberculose) em PBMRs que levaram ao financiamento sustentável e à capacitação. O *Africa Health Research Institute* (https://www.ahri.org/) e a INDEPTH *Network* (https://www.indepth-network.org/) são exemplos de iniciativas comprometidas com a descolonização da pesquisa em saúde. As redes de pesquisa em UTIs são movimentos transformadores e prósperos em que pesquisadores de PARs e PBMRs podem colaborar globalmente e se beneficiarem mutuamente, demonstrando, em última instância, que os esforços coletivos superam as contribuições individuais. Ao ampliar as vozes e o conhecimento especializado dos pesquisadores em PBMRs, podemos garantir que a comunidade global de cuidados intensivos se beneficie de diversas percepções e soluções adaptadas a diferentes contextos. Um ponto-chave para o sucesso é adotar os princípios levantados pelo movimento #DecolonizeScience, garantindo que os pesquisadores e parceiros do sul global tenham um lugar real na mesa, desde a concepção e a priorização da questão da pesquisa até a autoria. O valor do financiamento alocado para as instituições de PBMRs é crucial nesse processo, cobrindo não apenas o estudo, mas também o treinamento da equipe local, com o objetivo de deixar nesse país uma melhor compreensão e habilidades em termos de liderança para a pesquisa.

**Figura 1:** Importância, desafios e possíveis soluções para preencher a lacuna de conhecimento sobre cuidados intensivos em países de baixa e média renda.

PBMRs - países de baixa e média renda; PARs - países de alta renda.

## BARREIRAS DE PUBLICAÇÃO, VIESES E "ACESSO ABERTO": O PAPEL DOS PERIÓDICOS NA MITIGAÇÃO DAS DESIGUALDADES E NO AUMENTO DE PUBLICAÇÕES DE PESQUISAS DE PAÍSES DE BAIXA E MÉDIA RENDA

Apesar de abrigar 84% da população mundial, há uma sub-representação substancial da pesquisa realizada em PBMRs.^(6)^ Isso ocorre por meio de uma proporção baixa ou insignificante de pacientes de PBMRs em grandes estudos internacionais, um pequeno número de estudos conduzidos majoritariamente em PBMRs ou por meio de estudos exclusivamente em PBMRs em que pesquisadores e instituições sediados em PARs conduzem a questão da pesquisa e lideram o trabalho intelectual. Essa situação cria uma grande lacuna de conhecimento que decorre de vários desafios. Em primeiro lugar, a predominância de periódicos em inglês cria uma barreira para os falantes não nativos de periódicos de PARs, em um mundo em que uma fração da população é falante nativa de inglês.^(7)^ Em segundo lugar, os custos de publicação representam uma barreira significativa, pois são um ônus importante para os pesquisadores de PBMRs com orçamentos limitados e com pouca ou nenhuma verba disponível para custos de publicação.^(8)^ Embora existam iniciativas, como a isenção de taxas de processamento de artigos para PBRs, isso exclui os PMRs. A desigualdade é evidente tanto na capacidade de financiar a pesquisa quanto na de financiar sua publicação em um cenário em que o "acesso aberto" é cada vez mais comum, pois está associado a altos custos de publicação. Por fim, evidências sugerem um viés no processo editorial e de revisão por pares,^(9)^ favorecendo estudos de centros acadêmicos baseados em PARs para publicação em periódicos de alto impacto.^(10)^ Esses vieses de publicação têm outras consequências. Como a inteligência artificial influencia cada vez mais a medicina, seus algoritmos aprendem com os dados existentes, o que pode perpetuar esses vieses quando aplicados aos cuidados críticos em ambientes de PBMRs.

O envolvimento total dos pesquisadores locais é fundamental e deve ser refletido em posições de autoria iguais. Como pesquisadores e editores de publicações médicas, desempenhamos papel fundamental na redução das desigualdades de publicação. A *Critical Care Science* tem o compromisso de fornecer uma plataforma para todos os países, independentemente da geografia, do idioma ou do nível socioeconômico. Nosso conselho editorial geograficamente diverso e o formato de acesso aberto total (gratuito para autores e leitores), com a eliminação das taxas de processamento de artigos, contribuem para essa missão de criar um refúgio seguro para publicações de pesquisadores de PBMRs, assegurando padrões científicos de alta qualidade. O modelo tradicional de acesso aberto é intrinsecamente falho. Ele pode oferecer isenções para os PBRs e ser aberto a leitores de todo o mundo, mas tem altos custos de assinatura para universidades e centros acadêmicos, além dos custos de publicação extremamente altos para pesquisadores de PMRs.

A mudança para um sistema de publicação imparcial, com revisão por pares justa e publicações científicas financeiramente acessíveis a todos, deve se tornar uma prioridade. A meta deve ser a adoção de escalas móveis ou publicações totalmente gratuitas. Isso exige um forte compromisso da academia, das sociedades médicas e dos financiadores. Essas são etapas cruciais para preencher a lacuna de conhecimento em cuidados intensivos e melhorar os resultados globais de saúde. Esta revista está dando um passo firme nessa direção. Incentivamos outras revistas a se juntarem à *Critical Care Science* nessa jornada, adotando práticas semelhantes e promovendo ativamente a pesquisa em PBMRs. Ao promover um cenário de pesquisa mais equitativo, garantiremos que os avanços tão necessários em cuidados intensivos melhorem a qualidade das evidências atuais e acelerem sua implementação internacionalmente, melhorando, em última instância, a prestação de cuidados e salvando vidas.
